# The vascular contribution to dementia: World Stroke Organization scientific statement

**DOI:** 10.1177/17474930261470506

**Published:** 2026-07-15

**Authors:** Geert Jan Biessels, Stephanie Debette, Fanny M Elahi, Fatemeh Geranmayeh, Gurpreet K Hansra, Timothy M Hughes, Masafumi Ihara, Jeremy D Isaacs, Hanna Jokinen, Alexi Nott, Sarah T Pendlebury, Ruth Peters, Julie A Schneider, Colin Smith, Eric E Smith, Prashanthi Vemuri, Joanna M Wardlaw, Alastair JS Webb, Donna M Wilcock, Atticus H Hainsworth

**Affiliations:** 1 Department of Neurology, University Medical Center Utrecht, Utrecht, The Netherlands; 2 Institut du Cerveau (ICM), Paris Brain Institute, INSERM U1127, UMR CNRS 7225 Paris, Sorbonne Université, Assistance Publique des Hôpitaux de Paris, Paris, France; 3 Department of Neurology, Icahn School of Medicine at Mount Sinai, New York, NY, USA; 4 Department of Brain Sciences, Imperial College London, London, UK; 5 Imperial College Healthcare NHS Trust, London, UK; 6 Centre for Healthy Brain Aging, Discipline of Psychiatry and Mental Health, School of Clinical Medicine, University of New South Wales Sydney, Sydney, NSW, Australia; 7 Department of Internal Medicine, Wake Forest University School of Medicine, Winston-Salem, NC, USA; 8 Department of Neurology, National Cerebral and Cardiovascular Center, Osaka, Japan; 9 Department of Neurology, St George’s Hospital NHS Foundation Trust and City St George’s, University of London, London, UK; 10 Division of Neuropsychology, Neurocenter, Helsinki University Hospital and University of Helsinki, Helsinki, Finland; 11 Department of Psychology, Faculty of Medicine, University of Helsinki, Helsinki, Finland; 12 Department of Brain Sciences, Imperial College London, London, UK; 13 Dementia Research Institute, Imperial College London, London, UK; 14 Wolfson Centre for Prevention of Stroke and Dementia, Nuffield Department of Clinical Neurosciences, University of Oxford, Oxford, UK; 15 NIHR Oxford Biomedical Research Centre and Departments of Medicine and Geratology, Oxford University Hospitals NHS Foundation Trust, Oxford, UK; 16 The George Institute for Global Health, Sydney, NSW, Australia; 17 Department of Pathology, Department of Neurological Sciences, Alzheimer’s Disease Research Center, Rush University Medical Center, Chicago, IL, USA; 18 Academic Department of Neuropathology, Institute for Neuroscience and Cardiovascular Research, The University of Edinburgh, Edinburgh, UK; 19 Department of Clinical Neurosciences, Cumming School of Medicine, University of Calgary, Calgary, AB, Canada; 20 Department of Radiology, Mayo Clinic College of Medicine, Rochester, MN, USA; 21 Institute for Neuroscience and Cardiovascular Research, Dementia Research Institute, The University of Edinburgh, Edinburgh, UK; 22 Department of Brain Sciences, Imperial College London, London, UK; 23 Imperial College Healthcare NHS Trust, London, UK; 24 Stark Neuroscience Research Institute, Indiana University School of Medicine, Indianapolis, IN, USA; 25 City St George’s, University of London, London, UK; 26 Department of Neurology, St George’s Hospital NHS Foundation Trust, London, UK

**Keywords:** Vascular cognitive impairment, vascular dementia, mixed dementia, Alzheimer’s disease, cerebral small vessel disease

## Abstract

**Background:**

Alzheimer’s disease (AD) and cerebrovascular pathology are the two most common causes of dementia, frequently co-occurring in older people. Community-based neuropathology studies indicate that vascular disease accounts for approximately one-third of the population-attributable risk of dementia, controlling for other pathologies (including AD). The proportion with vascular disease as co-pathology is likely to be higher (50–70%). The most common vascular substrate is cerebral small vessel disease, which includes small artery fibrosis (arteriolosclerosis), vascular amyloid deposits (cerebral amyloid angiopathy), and monogenic forms of small vessel disease, the commonest being Cerebral Autosomal Dominant Arteriopathy with Subcortical Infarcts and Leukoencephalopathy (CADASIL). Post-stroke cognitive impairment following both ischemic stroke and intracerebral hemorrhage also contributes.

**Aims and Methods:**

In this World Stroke Organization (WSO) scientific statement, we assembled a multi-disciplinary international group of experts to review the vascular contribution to dementia, encompassing both vascular and neurodegenerative dementia. This statement has been reviewed and approved by the WSO executive.

**Results:**

We summarize the epidemiology, neuropathology, cognitive profile, clinical impact and management of vascular disease in dementia and discuss the recent VasCog-2-WSO diagnostic criteria. We consider the substantial overlap with clinical stroke and with AD dementia. We catalog transcriptomic and proteomic studies that have revealed novel candidate molecules (*COL4A1/4A2, HTRA1, TRIM47, FOXF2*) as possible treatment targets. We appraise imaging-based biomarkers relevant to vascular disease and potential biochemical markers (vascular endothelial growth factor-A, placental growth factor, interleukin-6, matrix metalloproteinase-9, cathepsin-B). We highlight the potential for vascular interventions to treat not only vascular dementia but also the vascular component of neurodegenerative dementia. We review recent clinical trials targeting multiple pathways, including nitric oxide signaling, high blood pressure, the GABAergic system, angiogenic activity, microglial inhibition, PDE3 and PDE5 inhibition, as well as dietary supplementation with omega-3 fatty acids, s-equol and vitamin E. Finally, we consider upcoming opportunities and challenges relevant to vascular disease in dementia.

**Conclusion:**

The vascular contribution to dementia is i) substantial, ii) increasingly understood at molecular and mechanistic levels, iii) a source of potential treatment opportunities.

**Data Access Statement:**

no original data are presented in this document.

## Graphical abstract

**Graphical abstract:**
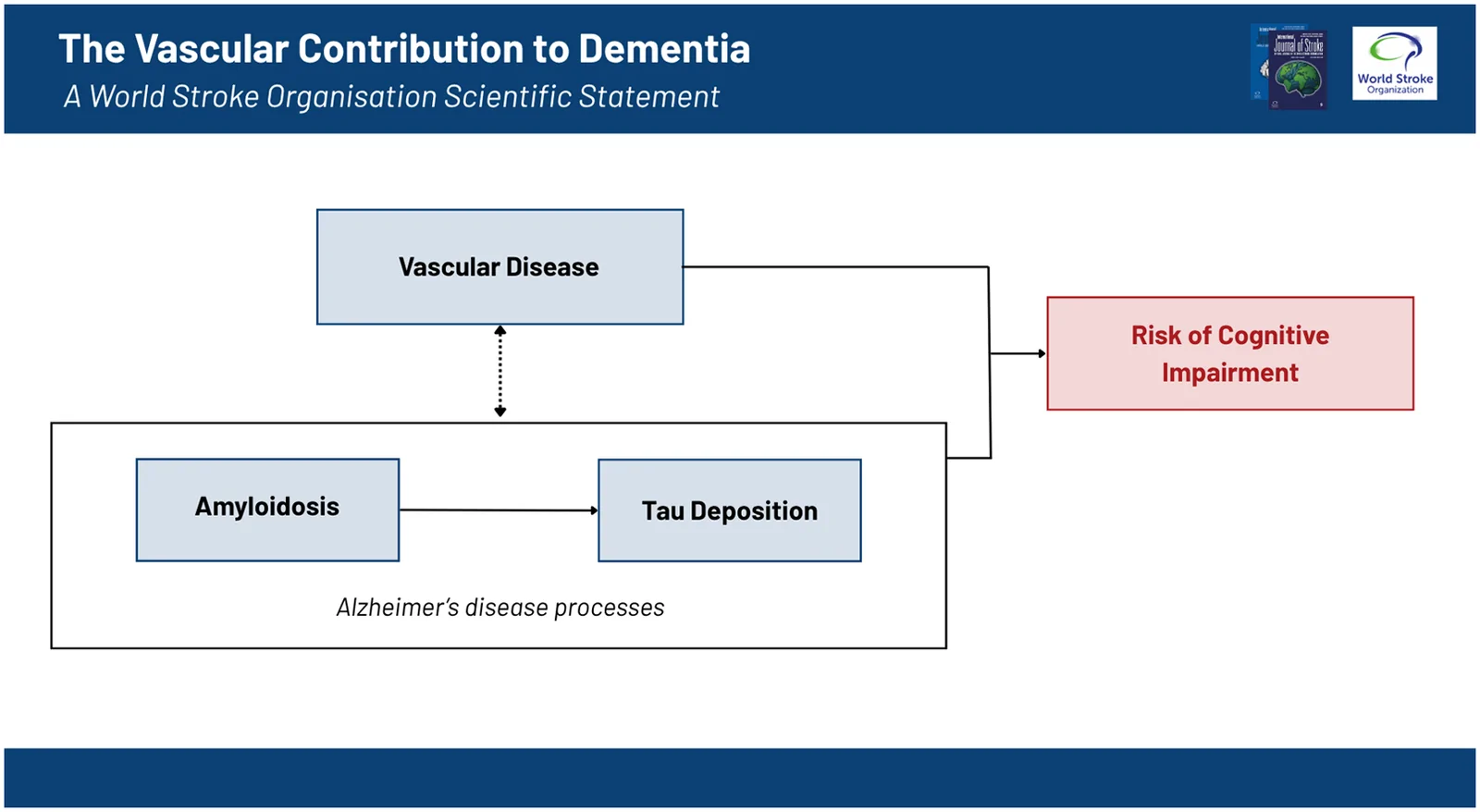

## Introduction: vascular disease in dementia

Dementia is a major cause of 21st-century human morbidity, and vascular disease is a major source of dementia. It is now apparent that various forms of cerebrovascular disease play a significant role in cognitive decline in older people and are at least partly responsible for cognitive impairment in the majority of people with dementia.^1–3^ Large neuropathological studies of autopsy brains overwhelmingly indicate that most clinical dementia is “mixed dementia,” driven by a combination of Alzheimer pathology and vascular disease (definitions are given in Box 1).^4–8^

**Box 1.** Definitions.

Vascular dementia (VaD) is a clinical diagnosis of dementia that is primarily caused by cerebrovascular disease. The observed cognitive state is temporally related to the occurrence of cerebrovascular injury (such as cumulative SVD or a clinical stroke event) or neuroimaging evidence of covert vascular brain injury, and is not sufficiently explained by Alzheimer’s or other neurodegenerative pathology. At least 8–10% of primary dementia diagnoses,^3,18^ VaD is a substantial source of clinical dementia. Post-stroke cognitive impairment (PSCI) is defined as any degree of impairment in one or more cognitive domains occurring after stroke, whereas post‑stroke dementia (PSD) refers to cognitive impairment severe enough to interfere with independence in daily functioning. Mixed, or multiple etiology, dementia refers to the common scenario where multiple neurodegenerative or vascular etiologies are present and contribute to dementia. Autopsy studies suggest that the combination of AD pathology and vascular disease is the single most common neuropathological correlate of later life dementia.^8^ Vascular cognitive impairment (VCI)^25^ is an umbrella term for all types, mechanisms and severity of vascular-related cognitive impairments, including mild, domain-specific deficits and dementia. VCI refers to a syndrome of cognitive impairment with evidence either of clinical stroke or of subclinical vascular brain injury, encompassing all the cognitive disorders associated with cerebrovascular disease, from mild cognitive deficits to frank dementia, also in the context of mixed etiology.^25,26^ Vascular contributions to cognitive impairment and dementia (VCID). This term was proposed to embrace all vascular aspects of dementia,^26,27^ from molecular causes through pathological features and biomarkers, to clinical symptoms and care pathways. VCID has proved a useful concept for research studies, funding schemes and conference programs. This broad concept embraces cognitive decline resulting from vascular disease of any type (not limited to brain vascular disease), also in the context of mixed etiology. More recently, a clinical diagnosis of Vascular Cognitive Impairment and Dementia has been proposed, also assigned the acronym VCID (see Box 2).^2^ The VasCog-2-WSO criteria subdivide VCID into three stages: at-risk VCID, mild VCID, and major VCID. This definition takes into account preclinical/at-risk VCID to allow early recognition of cognitive decline in patients with vascular etiologies. The criteria recognize the importance of neuroimaging in supporting a clinical diagnosis of VCID, or the presence of a genetic predisposing disorder such as CADASIL.^2^ Alzheimer’s disease (AD). Agreement on this term is elusive.^28,29^ AD neuropathology refers to a combination of neurofibrillary tangles within neurones and extracellular amyloid deposits (as reported by Dr Alzheimer). AD dementia refers to a clinical diagnosis of dementia based primarily on characteristic clinical features and neuropsychological findings, assumed to be caused by AD pathology. Diagnostic certainty has increased with the growing availability of in vivo markers for AD pathological change, including fluid biomarkers such as hyperphosphorylated tau and amyloid-β, as well as positron emission tomography (PET) imaging. Building on these advances, a biological definition^30^ of AD has been proposed, based on core biomarkers or neuropathological evidence of β-amyloid deposition (A), tau hyperphosphorylation (T) and neuronal death (N).^29^ The ATN proposal gave little consideration of co-morbid vascular disease in AD,^29,30^ prompting a response from some vascular researchers.^31^ The scheme was later modified^32^ to acknowledge the importance of infarcts and WMH of presumed vascular origin as contributors to cognitive decline.

A compendium of neuropathology, genetics, neuroimaging and clinical data now give us two clear lessons. First, the vascular diseases that contribute to dementia are many and various. These include sporadic small vessel disease (SVD) encompassing arteriolosclerosis, vascular amyloid deposits and micro-atheroma,^5,8–11^ often seen as white matter hyperintensities (WMH);^5^ intracranial atherosclerosis; large vessel infarcts or hemorrhagic lesions;^12^ and monogenic forms of SVD. These monogenic forms include Cerebral Autosomal Dominant Arteriopathy with Subcortical Infarcts and Leukoencephalopathy (CADASIL), Cerebral Autosomal Recessive Arteriopathy with Subcortical Infarcts and Leukoencephalopathy (CARASIL) and Collagen type-4 *COL4A1/COL4A2* mutations.^10,13,14^ The second lesson is that the main form of vascular disease in dementia, in terms of abundance and population impact, is sporadic SVD (often silent, or covert SVD). Neuropathological studies make clear that post-stroke cognitive impairment represents a relatively small proportion of the contribution of vascular disease to dementia.^4–7,15^

Due to its widespread occurrence and strong association with dementia risk, vascular disease offers viable therapeutic targets in dementia.^16,17^ This concept is supported by large epidemiological studies (see a recent review^18^) where potential vascular factors figure prominently among the top-line dementia risk drivers: physical inactivity, smoking, hypertension, high LDL-cholesterol, obesity, and high alcohol consumption.^19^ This claim is further strengthened by recent large clinical studies (SPRINT-MIND; China-Villages; US-POINTER)^20–22^ where interventions targeting cardiovascular risk gave significant benefit with respect to cognitive outcomes. This evidence has shaped policy and guidelines for blood pressure control^23^ considering brain health and dementia risk.^24^

This multi-disciplinary team aimed to provide a statement on the state of the art in vascular disease in dementia that has emerged in the past decade. The text is co-written by 20 international experts selected by the corresponding author to provide diversity across gender (11 female, 9 male) and nationality (Australia, Canada, Finland, Japan, Netherlands, France, the United Kingdom, and the United States). The draft structure was written by the corresponding author. Each section was drafted by a lead author, then updated by a small team of subject experts (2–5 in number). Sections were collated by the corresponding author and circulated for input by the full author team. Citations are derived from their collective expert knowledge and bibliometric searches.

## Epidemiology

Estimates of the proportion of clinical dementia cases that are reported to be driven, completely or in part, by vascular disease vary widely.^18^ In three North American community-based neuropathology studies^18^ cerebrovascular disease accounted for approximately one third of the population attributable risk of dementia, controlling for other pathologies (including AD).^33–35^ The proportion with vascular disease as co-pathology is likely to be higher.^5,7,8^ Some estimates put the prevalence and incidence of cases with vascular contributions as high as 50–70% depending on the population composition, ethnicity, risk factor exposure and age.^5,18^

Deriving definitive estimates of dementia cases that are solely driven by vascular pathology, or weighing the importance of a vascular component, is challenging due to variation in definitions, breadth of vascular diseases, availability of imaging data and varied population characteristics in observational studies. Current estimates are likely an underestimate, given the high prevalence of cardiovascular risk factors.^18^

Understanding of heterogeneity of dementia pathology and risk factors has grown substantially over the last 30 years,^36^ with a range of potential risk factors identified in early, mid and later life.^19^ These incorporate individual lifestyle behaviors (tobacco smoking, alcohol consumption, physical activity, social engagement), health-related measures (Body Mass Index, midlife hearing loss), clinical factors (high blood pressure, diabetes mellitus, hypercholesterolemia), socioeconomic drivers (access to education, early life adversity) and population exposures (air pollution).^19,37^ Many of these are established risk factors for cardiovascular disease, stroke, and SVD.^38^ The combination of a long prodromal period, key risk factors at different life stages and likely interaction between risk factors all highlight the importance of a life course risk reduction approach, and the need to focus on modifiable risk.^39^

Although a globally aging population means that dementia prevalence will continue to rise, since a substantial number of dementia risk factors can be considered as cardiovascular,^19^ there are consequent opportunities to reduce risk. Aligned to this, several studies have shown falling incidence^40,41^ in step with falling mortality for cardiovascular diseases, including stroke, and considered to be due to successful targeting of cardiovascular risk. These include blood pressure control and cholesterol lowering, alongside public health campaigns and legislation to reduce smoking and alcohol consumption both in general and at-risk populations.

At a population level, the Lancet commission on dementia prevention, intervention and care (2024) uses a population attributable fraction (PAF) approach to identify key risk factors for dementia.^19^ The report provides a summary of the current evidence and estimates the proportion of dementia cases that could theoretically be prevented if these risk factors were eliminated. Importantly, the relative contribution of individual risk factors differs between population groups,^42^ and may change as the data are updated. For example, groups experiencing greater social disadvantage, lower access to healthcare and poorer diet have higher rates of cardiovascular disease and poorer management/control, resulting in a greater proportion of dementia attributable to cardiovascular risk factors.^19^ Limitations in current estimates arise from a lack of data on representative population groups and the inherent difficulties in attributing dementia type (particularly in older datasets or where imaging is not available). These limitations potentially drive an underestimate of the impact of vascular risk factors, since underrepresented or excluded populations are likely to include those at higher cardiovascular risk, including those with prior stroke. Historical data may also inflate the role of AD, where the diagnostic threshold for vascular dementia required evidence (e.g. of prior stroke, or imaging evidence of brain infarction or WMH), but where resource constraints made this difficult to obtain.

There is increasing consensus for population-level interventions to reduce exposure to dementia risk factors.^43^ In terms of interventions for individual patients, optimal management of cardiovascular risk factors, in particular hypertension^21^ and diabetes,^44^ have now also been shown to reduce progression to MCI and dementia. There remain important questions as to whether multidomain interventions (encompassing, for example, increased physical, social and cognitive activity, Mediterranean diet adherence, better sleep hygiene) reduce incident dementia, as opposed to, or in addition to, improving cognitive test scores in cognitively unimpaired individuals.

## Pathologic changes associated with vascular disease

Large clinical neuropathological studies within community cohorts have greatly advanced understanding of vessel pathology and the associated vascular lesions underlying dementia.^4–7^ The consistent message is that a focus on post-stroke cognitive decline (due to incident stroke lesions),^45,46^ greatly underestimates the role of vascular disease in dementia.^11,18,46^ Specifically, there is abundant subclinical vessel disease and vascular tissue injury that has been shown in multiple studies^5,8,36,47^ to contribute to cognitive decline and dementia in old persons without a known history of overt stroke.

There is a range of vascular pathologies that can be associated with cognitive impairment.^8,9,36,48^ These include those of larger vessels (atherosclerosis) and smaller vessels (arteriolosclerosis and cerebral amyloid angiopathy). Enlarged perivascular spaces can also be seen on brain specimens and are noted in imaging as another vessel pathology related to cerebrovascular risk. It is apparent that in aging, the most prevalent and likely the most globally impactful, substrates of cognitive impairment are microvascular (“small vessel disease”; SVD).^5,6,10,11,49^

The term “small vessel disease” is used in clinical, imaging and autopsy studies, but the boundaries of this term can be a little unclear since it often refers to a conglomerate of pathologic and radiologic findings including silent lacunes, covert cortical or subcortical infarcts, microinfarcts, diffuse WMH and microbleeds. The most common forms of SVD in the brains of older people are small artery fibrosis (arteriolosclerosis)^5^ and vascular amyloid deposits (cerebral amyloid angiopathy, CAA).^50^ Arteriolosclerosis is a fibrotic thickening of small penetrating arteries, seen in subcortical white matter and deep gray nuclei,^5^ and is associated with hypertension and aging.^38^ Arteriolosclerosis is a major cause of diffuse white matter damage (radiologically, WMH), small and lacunar infarcts, microhemorrhages and microinfarcts.^5^ CAA is the deposition of beta-amyloid around penetrating and leptomeningeal arteries (and in some brains, capillaries),^48,50^ often but not always accompanying AD pathology.^51,52^ CAA typically spares deep white matter penetrating arterioles,^50,51^ which are the vessels most affected by hypertensive SVD. Both subcortical arteriolosclerosis and cortical CAA are often seen together in the aging brain, though their risk factor profiles differ: arteriolosclerosis is primarily associated with traditional vascular risk factors, whereas CAA shares many of the risk factors implicated in AD-type dementia. Nonetheless, their downstream consequences are similar. Arteriolosclerosis and CAA both cause cerebral microbleeds, the former in subcortical areas, CAA primarily in cortical gray matter. Microbleeds at both locations are associated with cognitive impairment and with risk of larger intracerebral hemorrhage.^50^ As with neurodegenerative pathologies that are often mixed, vascular pathologies commonly occur together such that the brains of older persons typically exhibit two or more vascular diseases and multiple types of tissue injuries.^8,48,53^ Intracranial atherosclerosis^54^ also contributes to tissue injury and dementia risk. Indeed, atherosclerosis may affect penetrating vessels. Both arteriolosclerosis and atherosclerosis contribute to cortical and hippocampal atrophy.^55^ With this in mind, the complexity of vessel pathology and tissue injury goes even beyond the likely global impact of SVD.^5,6,10,11,49^

The pathology of CAA is well reviewed^48,50^ and reproducible assessments exist both for severity^56^ and extent^57^ of the vascular disease, supporting a standardized approach to research analysis. A recent study^52^ highlighted the variability of CAA across those with no, low, and high AD pathology. By contrast, it is notable that arteriolosclerosis severity and arteriolosclerosis association with vascular risk factors may vary across brain regions.^49^ This further complicates comparative analysis across research studies.^53^ Assessing the true role of arteriolosclerosis in vascular cognitive decline will require standardized approaches to the descriptions of the vascular pathology and the downstream substrates such as lacunar infarcts and white matter rarefaction, and how this relates to WMH. Other substrates for VCID may be directly or indirectly related to arteriolosclerosis (enlarged perivascular spaces, clearance mechanisms) but are currently not included in estimates of the prevalence of SVD pathology.

## Cognitive impact of vascular disease

Vascular disease can impact a variety of cognitive domains depending on the underlying lesions and lesion locations. Symptoms caused by SVD are wide-ranging and may affect any of the major cognitive domains, even without overt dementia or stroke.^58^ Processing speed, attention and executive functions are the most prominently affected cognitive domains in SVD.^2,59,60^ Specifically, timed cognitive tests sensitive to these domains have had the strongest association with SVD neuroimaging markers.^59^ When tested in cohorts of SVD, all cognitive domains are known to be affected, including language and visuospatial functioning^58^ (Figure 1).

**Figure 1.**
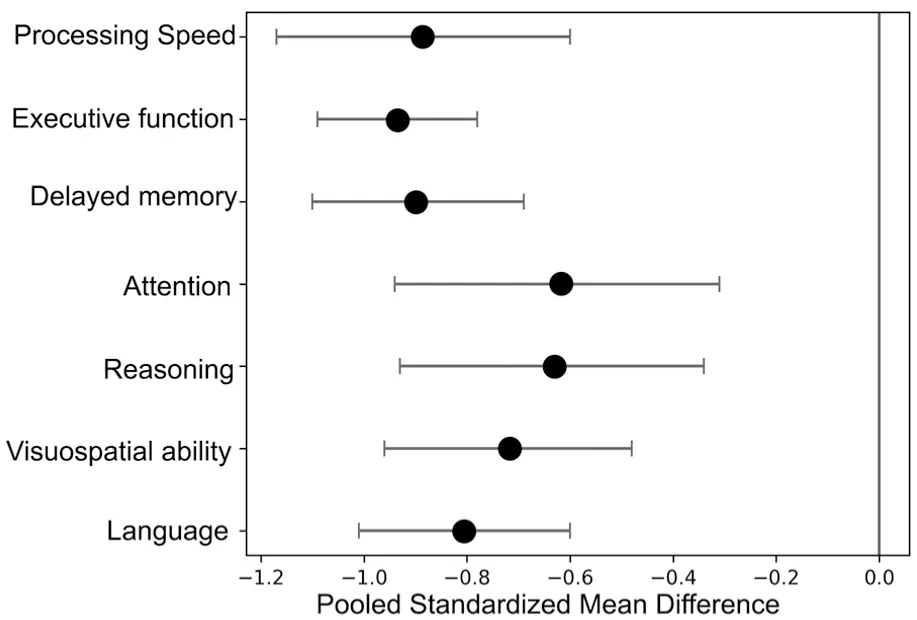
Forest plot showing pooled standardized mean differences (SMDs) for each cognitive domain in cohorts with SVD compared to controls. Points represent pooled effect estimates and horizontal lines indicate 95% confidence intervals. Negative values indicate poorer performance in the patients with SVD group relative to controls. Within the SVD cohorts, those with a cognitive presentation of SVD performed more poorly than cohorts with covert SVD on tests of delayed memory, executive function, and visuospatial ability. They also performed more poorly than cohorts with stroke presentations of SVD on tests of delayed memory. Adapted from meta-analysis of published data from 69 studies by Hamilton et al.^58^

Common co-occurring neuropsychiatric manifestations such as apathy, depression, and anxiety^58^ exacerbate the associated cognitive impairment,^61^ whereas high cognitive reserve may protect against deterioration.^62,63^ The most rapid rate of decline is observed in individuals presenting with mixed pathology that includes cerebrovascular disease at autopsy.^1^ In patients with both AD and vascular pathology, cognitive impairment is likely to be more global, further diminishing daily functional abilities due to limited compensatory mechanisms.

Some form of objective cognitive evaluation is recommended for all patients with significant cerebrovascular imaging findings and suspected cognitive impairment. Cognitive screening tools are quick and simple to use but limited in sensitivity, particularly at the early stages of decline, although more sensitive, scalable assessments specifically for cerebrovascular disease are emerging.^64^ Neuropsychological assessment is key to determining the pattern and severity of impairment in relation to individual premorbid cognitive functioning. Although there is no consensus on the specific cognitive instruments to use in the context of clinically manifest stroke, screening should include assessment of domain-general (executive function, attention and reasoning) as well as domain-specific cognitive functions (language, visuospatial attention and memory) as well as assessment of functional outcomes. Recently, harmonized neuropsychological assessment guidelines for VCID have been updated, including recommendations for core and comprehensive batteries, cognitive screening, tele-health and computerized methods, as well as assessment of diverse populations.^60^

## Clinical diagnosis, clinical impact

In the updated VasCog-2-WSO diagnostic criteria^2^ (Box 2), a diagnosis of “vascular cognitive impairment or dementia” (VCID) rests on three pillars. These are (1) an acquired cognitive deficit, (2) a significant burden of cerebrovascular disease, and (3) a likely causal relation between the cognitive deficit and the vascular disease. In clinical practice, the diagnostic process may evolve along different pathways, depending on the initial symptoms of the patient.

**Box 2.** The VasCog-2-WSO clinical diagnostic criteria.

The inherent heterogeneity of vascular disease and the cognitive consequences represent a challenge for establishing clinical diagnostic criteria. The International Society of Vascular Behavioral and Cognitive Disorders (VasCog)—World Stroke Organization (WSO) criteria were updated in 2025.^2^ These update the VasCog-1 criteria to resolve inconsistencies with terminology and to align with advances in neuroimaging and biomarkers. Given the importance of preventive intervention, the VasCog-2-WSO criteria^2^ have introduced a preclinical category, aimed at identifying persons who are asymptomatic for cognitive impairment but present with significant cerebrovascular pathology (identified via neuroimaging). The framework recognizes ischemic and hemorrhagic subtypes of VCID, but identifies aetiological subtyping as a topic that needs further development. Preclinical/At-Risk VCID. Both 1 AND 2 are required: 1. Neuroimaging evidence of at least ONE of the following: a. Multiple infarcts or single extensive infarct. b. Multiple lacunes (⩾2) outside the brainstem or 1 lacune if it is strategically located with WMH. c. Extensive and confluent WMH. d. One large intracerebral hemorrhage if strategically placed or ⩾ 2 intracerebral hemorrhages. 2. Does not meet the criteria of mild or major cognitive impairment/dementia with no concerns of cognitive change by the person, their informant or clinician. Vascular MCI (Mild severity VCID). Both 1 AND 2 are required: 1. Evidence of modest deficits on objective cognitive assessments in ⩾ 1 cognitive domain (Attention and processing speed, Executive function, learning and memory, language, perceptual-motor function or social cognition). Test performance ranges 1–2 SDs below norm. Cognitive deficits not sufficient to significantly impair instrumental activities of daily living. 2. Evidence for Predominantly Vascular Etiology of Cognitive Impairment: The onset of the cognitive deficits is temporally related to clinical strokes, or there is neuroimaging evidence of substantial cerebrovascular disease. Vascular Dementia (Major severity VCID). Both 1 AND 2 are required: 1. Evidence of substantial cognitive decline in ⩾ 1 cognitive domain. Test performance ⩾ 2 SDs below norms. Separate from physical deficits, cognitive deficits are sufficiently substantial to interfere with activities of daily living. 2. Evidence for Predominantly Vascular Etiology of Cognitive Impairment.

In patients who present with an acute stroke event, the initial focus in diagnosis, treatment, and care is on the causes and acute consequences of that event, mostly offered in the setting of a stroke care pathway. The etiology of the vascular disease is investigated through established diagnostic criteria. This etiological diagnosis informs prognosis (such as the risk of stroke recurrence) and directs secondary prevention. With regard to VCID in this setting, the challenge is not to overlook the cognitive consequences of stroke. Because of the hyperacute nature of stroke, cognitive consequences may initially be unnoticed and may become evident only when the patient is released from intensive hospital care and tries to resume her or his activities of daily life. Because cognitive deficits are so common after stroke, high vigilance is warranted. Recently, the European Stroke Organisation (ESO) Dementia Committee offered guidance on the recognition of and vigilance for cognitive deficits after stroke.^65^

On the other hand, patients who primarily present with cognitive complaints are typically evaluated in a Memory clinic setting. Here, the challenge is not so much in recognizing the cognitive deficit, but in the proper identification and classification of cerebrovascular disease, particularly in patients who never experienced stroke-like symptoms. Here, the commonest form of cerebrovascular disease encountered is SVD, often in combination with other pathologies, particularly AD. In most of these cases, the presence of SVD will primarily be derived from brain imaging, on CT or preferably MRI scans.^66^ Because manifestations of SVD are so common on brain MRI in people over the age of 60, it can be difficult to judge whether a given amount of visible lesions (particularly WMH) can still be regarded as “normal” for age, or should be regarded as “too much” and therefore a likely contributor to the cognitive deficit. The recent publication of normative age- and sex-specific WMH volumes, derived from a pooled analysis of 15 population-based cohorts, provides a valuable guide.^67^

Another issue in this setting is how to optimize vascular prevention, including balancing risk and benefit of antithrombotic medication in patients with multiple microbleeds.^68^ An ESO Guideline^69^ recommendation is not to start such antithrombotic medication solely in response to detecting otherwise-covert SVD on brain imaging (i.e. WMH) since evidence indicates that antiplatelet drugs worsen outcomes in WMH if there is no other good reason to prescribe them (like ischemic heart disease or history of clinical ischemic stroke), but to optimize vascular risk management in all other respects in all cases.^69,70^ Detailed information, with a review of the evidence of management of SVD in this setting, can be found in two recent ESO guidelines.^69,70^

## Overlap with AD

The combination of vascular disease and AD pathology is the most common neuropathological correlate of dementia.^71^ The frequent association of these two pathologies has generated hypotheses that they are pathophysiologically connected.^31,72^ The most direct connection between AD pathology and cerebrovascular pathology is via vascular amyloid deposition leading to CAA.^50,73^ AD pathology is usually accompanied by at least some degree of CAA. Independent of the amount of AD pathology, more CAA pathology is associated with worse cognitive function, indicating that CAA is not a mere bystander but is itself accelerating cognitive decline.^74^

This close relationship between AD pathology and vascular disease raises the question of whether vascular disease causes or accelerates cognitive decline. In the National Alzheimer’s Disease Coordinating Center (NACC) study and Religious Orders Study/Rush Memory and Aging Project, atherosclerosis of the intracranial arteries was associated with higher AD pathology, controlling for age.^54,75^ However, in vivo imaging studies in Atherosclerosis Risk in Communities suggest that carotid and intracranial measures of atherosclerosis^76,77^ are not generally significantly associated with amyloid deposition, where association may be stronger in APOEe4 carriers. In a large population-based epidemiological study, midlife vascular risk factors predicted elevated amyloid PET signal later in life.^78^ There is also some evidence for the interaction between vascular risk factors and tau pathology.^79–82^ In contrast, the severity of arteriolosclerosis was not associated either with amyloid plaques or with neurofibrillary tangles in a large, pooled analysis of six brain banks.^83^

Sporadic AD is thought to result from failure to clear beta-amyloid peptides,^84^ prompting intense interest in the mechanisms by which fluid and solutes enter and exit the brain, including the roles of vascular pathways. A decline in cerebral blood flow is among the earliest detectable changes in persons with AD.^85^ Two hypothesized routes for fluid clearance from the brain parenchyma are a peri-arterial pathway, in which fluid exits along the basement membrane of arteries,^86^ and the glymphatic pathway, in which fluid exits along perivenous routes.^87^

Recognition of cerebrovascular and AD co-pathology is increasingly important with the emerging amyloid-targeting therapies, such as lecanemab and donanemab, that cause amyloid-related imaging abnormalities (ARIA).^88,89^ ARIA-edema (ARIA-E) and ARIA hemorrhages (ARIA-H) have been reported across anti-amyloid clinical trials and may occur together, often early, during the treatment.^89^ Most ARIA cases are asymptomatic, and those that are symptomatic often occur at higher doses and resolve within 3–4 months or upon treatment cessation. Fatal cases have revealed severe CAA with vascular and perivascular immune responses, suggesting that interactions among beta-amyloid, amyloid-targeting therapies, the immune system and blood vessels play a role in causing these serious adverse events. *APOE* haplotype, hypertension, micro-hemorrhages at baseline and increasing treatment dosage are major risk factors for ARIA.^88^

An important question remains as to whether AD pathology and vascular pathology are synergistic in their effects on cognition, or simply additive. True biological synergy—a combined effect greater than the sum of individual effects–is difficult to demonstrate with confidence in any experimental system. This is likely to be all the more challenging in noisy cognitive data from older humans. Some studies have supported synergy^90,91^ between AD and vascular lesions, others simple addition.^92–95^ The degree of synergy, if present, appears likely to be modest.^96,97^

In summary, the combination of AD and vascular disease, converging to produce brain injury and dysfunction, is the most common pathological background of dementia. Emerging evidence suggests a bidirectional relationship whereby AD causes vascular damage (via CAA) and vascular disease promotes the development or progression of AD.

## Overlap with clinical stroke

Stroke and dementia have a bidirectional relationship. Cognitive decline is associated with increased risk of stroke and of more severe stroke, while incident stroke increases risk of dementia.^98^ While the reasons for this bidirectional relationship are unclear, shared risks include vascular disease (especially in midlife), environmental, genetic and early life factors. Preventing stroke is therefore of paramount importance in reducing the population burden of dementia.

Following a manifest stroke, depending on the population studied, 40–80% of patients have impairment in at least one cognitive domain, although some recovery in the first 3–6 months is common.^99^ The 5-year risk of post-stroke dementia is as high as 80% in the older patient population with more severe strokes.^12^ Around 10% of patients with stroke have pre-existing dementia and the prevalence increases linearly with event severity.^100^ Of those who are dementia-free, 10% with first-ever stroke and at least 30% of those with recurrent stroke, will develop dementia within the first year. Dementia risk after stroke is front-loaded, with the highest risk in the first year, attenuating thereafter. Compared to the age and sex-matched background population, the incidence of dementia in the first year after stroke is elevated 50× after severe stroke (NIHSS > 10) but falls to around 6.5× thereafter.^12,98^ Although the risk after transient ischemic attack (TIA) is lower, it is nevertheless 3.5 times higher than that of the background population in the first year. This has the effect of bringing forward the onset of dementia at the population level by around 2 years for TIA, 4 years for minor stroke and 15 years at least for major stroke.^12,101^

Ischemic and hemorrhagic strokes are associated with similar risks overall after correction for stroke severity.^12,102^ The risks are higher after lobar hemorrhage secondary to CAA than after deep hemorrhage of hypertensive etiology. This differential risk is likely mediated by coexisting AD pathology.^103^

Risk factors for dementia after stroke can be divided into those relating to pre-existing brain vulnerability and those relating to the impact of the stroke lesion (volume, topology, and number of lesions).^12^ Important covariates include age, and initial stroke severity, while vulnerability factors include older age, female sex, low education, racial minoritization, low socioeconomic status, prior cognitive decline, previous stroke, SVD and cerebral atrophy. Among SVD manifestations on neuroimaging (lacunar infarction, WMH, microbleeds, enlarged perivascular spaces),^66^ WMH load is most strongly related to post-stroke dementia.

Brain atrophy, both global and in specific locations (including focal temporal lobe atrophy), is associated with post-stroke dementia.^104,105^ Focal temporal lobe changes suggest pre-existing neurodegeneration, specifically AD, as do associations with APOE genotype. APOEe4 is the strongest sporadic risk factor for AD and is also a risk factor for poor recovery after head injury. APOEe4 is a well-established risk factor for sporadic CAA, whereas its independent association with non-amyloid sporadic SVD is inconsistent. APOEe4 homozygosity is associated with post-stroke dementia even after adjustment for stroke severity and coexisting SVD.^106^ Associations are stronger for dementia after major stroke than after TIA or minor stroke, supporting a role for increased susceptibility and reduced brain reserve in ε4/ε4-associated early post-stroke dementia, in keeping with neuropathological evidence that subclinical AD may be revealed by a stroke event. Further, ε4/ε4 may worsen the impact of the stroke lesion through amyloid-associated post-stroke inflammation.^106^

Dementia is more likely after multiple strokes, strokes in the left hemisphere and stroke with aphasia.^104^ Stroke lesion location is a major determent of the cognitive outcome. Multicohort lesion mapping studies have identified specific strategic locations including left frontotemporal lobes, left thalamus, right parietal lobe and cholinergic pathways, as most associated with PSCI.^104^

Delirium, infection and frailty are more recently identified, and potentially modifiable, independent risk factors for dementia. Delirium is associated with dementia in both post-stroke and non-stroke cohorts,^107^ and accelerates cognitive decline in AD. The impact of delirium appears greatest in previously robust, dementia-free individuals.^107,108^ Infection increases the risk predominantly of vascular dementia, is associated with vascular pattern cognitive deficits, and appears to require the presence of SVD to exert its effect.^107,109^ The mechanisms underpinning the relationships of delirium and infection with future dementia are unclear, but increased permeability of the blood-brain barrier (BBB) facilitating entry of systemic factors to the brain may be a factor. Precipitated or accelerated cognitive decline may result from effects on activated microglia, participating in pre-existing neurodegeneration. Alternatively, SVD may be exacerbated by peripheral inflammatory mediators.^110^ Frailty is a risk factor for delirium and dementia, but relationships with specific dementia subtypes are unclear.^108,111^ Systemic comorbidities including diabetes, dyspepsia, osteoporosis and pain have also been linked to dementia, but there is a lack of data on links with specific dementia subtypes.^112^

In summary, maintenance of pre-stroke brain health, optimal primary and secondary stroke prevention, and acute stroke interventions to reduce stroke severity will reduce the population burden of dementia, including in vulnerable individuals with pre-existing AD pathology. Prevention and treatment of secondary insults, including delirium and infection post-stroke, may reduce stroke cognitive impacts, particularly in those with frailty and comorbidity.

## Molecular processes

Technological and bioinformatic advances have yielded rich novel data on molecular candidates in the vascular processes linked to dementia. Hundreds of DNA variants increase the risk of SVD in genome-wide association studies (GWAS) of small vessel stroke^113,114^ and clinical imaging markers of SVD, including WMH and perivascular space (PVS) burden.^115,116^ SVD is associated with both stroke and dementia and, similar to sporadic AD, is a heritable polygenic condition.^115,117^ In most dementia cases at the population level, SVD and AD pathology coexist. APOEe4 is the strongest genetic risk factor for AD and is also, although much more weakly, associated with SVD (specifically, increased WMH volume and BBB breakdown in SVD).^118^ GWAS for WMH of presumed vascular origin identified 27–31 genome-wide risk loci^115,119^ showing strong genetic correlation with blood pressure,^115,119^ as well as with stroke and AD dementia.^115^ Mendelian randomization studies show strong evidence for a causal relation of higher blood pressure with WMH volume (including among people without clinical hypertension) and of larger WMH volume with increased risk of stroke (ischemic and hemorrhagic), including multivariable analyses accounting for pulse pressure and interim stroke.^115,120^

Pathway enrichment analyses and colocalization of WMH GWAS variants with expression quantitative risk loci (eQTL) prioritized risk genes related to extracellular matrix and inflammation.^119,121^ A recent GWAS for PVS revealed 24 genome-wide risk loci,^116^ which similarly showed genetic correlation with stroke and blood pressure, including extracellular matrix genes, as well as genes involved in membrane transport and developmental processes.^116^ These studies revealed that risk variants for MRI markers of SVD in older age are already associated with subtle changes in brain structure in young adults in their twenties and enriched in genes predominantly expressed in fetal brain endothelial cells (BECs), pointing to lifespan processes,^115,116,122^ but also in other cell types from the neurovascular unit.

Single-cell gene expression studies indicate that many AD risk genes are highly expressed in BECs and fibroblasts^123^ (in addition to microglia/myeloid cells^124–127^) and are differentially expressed in disease.^128,129^ A subcluster of vascular mural cells was found to be enriched for extracellular matrix genes and was proposed to contribute to monogenic SVDs, including CADASIL, CARASIL and collagen IV deficiencies,^123^ all of which are extracellular matrix-associated pathologies. Similarly, WMH GWAS genes show consistent vascular differential expression in AD,^130^ which have been linked to extracellular matrix and basement membrane integrity (*COL4A1*/*COL4A2*), brain development (*FOXF2*, *ULK4*), cellular/metabolic functions (*SLC25A44*, *FAM117B*), inflammation (*PRKCH*), and vascular contractility and the cytoskeleton (*PLEKHG1*, *EVL*, *EPB41L3*, *CALCRL*).^123,128,129^ Vascular differential expression of PVS GWAS genes in dementia was related to basement membrane, adhesion and vascular stiffness (*LAMC*, *PPFIA1*, *CALD1*), gene regulation and specifically modulation of Tie2 signaling^131^ (*FOXF2*), cellular signaling, metabolism and stress (*PDE8A*, *ISYNA1*, *TP53RK*, *SLC20A2*, *SMIM19*) as well as astrocytic reactivity (*GFAP*).^123,128–130^ *APOEe4* was enriched for AD-associated gene expression changes in BECs, pericytes and fibroblasts.^128^ Future studies should examine vascular-associated changes in gene expression in the context of SVD and how these interact with AD-related dysregulation.

GWAS-derived variants are mostly noncoding and likely alter the function of gene regulatory regions called enhancers. Enhancers are extremely cell-type specific and distinguish the cell-type contribution of GWAS risk genes. The simultaneous profiling of gene expression and chromatin accessibility of vascular-enriched cell types (multiVINE-seq) has linked genes to genomic variants at distal enhancers.^132^ Consistent with prior findings, chromatin accessibility analysis implicated extracellular matrix proteins in vascular cell types for cardiovascular traits and inflammatory adaptor proteins in endothelial cells and microglia for AD risk.^132^ In parallel, epigenomic profiling of histone modifications of the major classes of brain cell types showed AD GWAS variants are strongly enriched at microglia enhancers,^124^ with a modest association for BECs.^133^ In contrast, SVD risk variants were highly enriched in enhancers of endothelial cells, mural cells (an umbrella term covering myocytes and pericytes) and astrocytes, implicating the vascular unit in SVD genetic susceptibility.^133^ Genes linked to distal GWAS variants at enhancers using chromosome conformation capture identified AD- and SVD-distinct genes, implicating amyloid and phospholipid processes in AD and senescence and extracellular matrix genes for SVD. For example, the SVD GWAS gene *TRIM47*,^115^ which has been associated with reduced expression in severe SVD and increased endothelial permeability,^134,135^ was epigenetically linked to BECs, mural cells and astrocytes. Large-scale transcriptomic and epigenomic studies with cellular resolution, ideally from brain white matter tissue and enriched in vascular cells, are needed to understand the gene regulatory mechanisms that are altered in SVD.

Plasma and cerebrospinal fluid proteomics have linked peripheral extracellular matrix and immune response proteins to brain imaging markers of SVD, including WMH and PVS burden.^136,137^ These included EPHB4 and TLR1,^136^ which were also identified as SVD risk genes in vascular cell types, and in microglia/myeloid cells and BECs, respectively.^133^ In addition, the SVD-associated proteins HTRA1, COL6A1, TLR1, and IL6^136^ have been linked to cardiovascular risk in microglia and vascular cell types.^133^ Additional CSF markers of extracellular matrix dysregulation and vascular remodeling were found elevated across multiple SVD manifestations, including ELN, POSTN, CCN2, and MMP12, further implicating endothelial and smooth muscle cells as key drivers of SVD pathology.^137^ A multi-trait analysis demonstrated that a subset of AD loci is shared with cardiovascular conditions.^138^ The overlap of genetic determinants of SVD with dementia subtypes and cardiovascular conditions remains to be fully examined to determine commonalities.

Genes and proteins with genetic-associations to disease have been used to prioritize repurposable drugs for AD,^133^ stroke,^114^ and SVD.^136^ Drugs have been prioritized for stroke using genetics, directional gene expression changes and causal associations through Mendelian randomization using protein quantitative trait loci,^114^ which highlighted F11, KLKB1, PROC, VCAM, GP1BA, and LAMC2^114^ as potential targets, of which the latter two specifically for small vessel stroke. Drug targets for SVD were further prioritized based on the association of plasma and CSF proteomics with SVD imaging markers, identifying EPO, LTF, TFPI, EPHB4, COL6A1, PCSK9, and GPNMB as candidate targets for drug repositioning for SVD.^136^ Drugs specific for some of these targets (EPO, PCSK9) are already in clinical use. Compounds that correct for gene expression changes of multiple putative microglia risk genes were identified by in silico screening of analogous cell lines in the LINCS CMap resource.^133^ This approach identified the vitamin D receptor agonist, calcitriol, as a potential therapeutic for microglia/myeloid cells in AD.^133^ This aligned with vitamin D deficiency as a risk factor for dementia, its anti-inflammatory role in immune cells and its increased activity in protective APOEe2-expressing microglia.^139^

## Neuroimaging markers

Imaging biomarkers for vascular disease represent an active area of research, yielding various measures of SVD, intracranial atherosclerosis, white matter diffusion metrics, and perfusion of cerebral blood flow. Recently published STRIVE2 criteria^66^ expanded and refined the standardization of lesion definitions on CT and MRI to improve consistency across studies. The most widely used imaging biomarkers for vascular disease in clinical trials and routine clinical practice are WMH, infarcts, microbleeds, and cortical superficial siderosis. While enlarged perivascular spaces^140^ have been tied to glymphatic dysfunction, these are typically not reported in routine radiological practice unless they are unusually prominent. All these markers have been linked to increased risk of stroke, mortality, and dementia.^15^

Diffusion MRI has been widely shown to capture white matter microstructural damage due to early vascular disease, before WMH lesions are visible on standard structural MRI sequences.^141^ Several composite imaging markers (including those proposed as part of the MarkVCID consortium)^142^ are currently based on diffusion MRI. Peak width of skeletonized mean diffusivity (PSMD)^143^ is a widely utilized global measure of white matter damage. Other MRI-based SVD surrogates have been proposed, including Arteriolosclerosis (ARTS)-Score^144^ for in vivo measurement of arteriolosclerosis, and cerebrovascular reactivity (CVR) for measuring physiological function of the brain microvasculature. MRI-based measures of intracranial atherosclerosis with vessel wall imaging show associations with incident cognitive impairment and dementia, independent of stroke^145^ and SVD.^146^ Measuring BBB permeability has also been proposed, with contrast agent leaking through the BBB considered reflective of early small vessel dysfunction.^147^ Newer MRI methods for measuring BBB dysfunction without contrast are also being developed and validated^148^ (based on other measures than gadolinium leakage).^148^ Intracranial atherosclerotic disease can lead to cognitive impairment through reduced blood flow and inflammation in downstream parenchyma.

Recent evaluation of these newer vascular markers in conjunction with AD biomarkers highlights the overlap and importance of vascular pathways in reducing the risk of cognitive impairment.^149,150^ There are exciting new horizons ahead as these markers are evaluated across different populations and slowly incorporated into clinical practice and clinical trials.

## Fluid biomarkers

Fluid-based biomarkers have a number of advantages. First, they are relatively inexpensive compared to neuroimaging; second, they are widely accessible since blood samples can be collected anywhere and shipped to laboratory sites for testing, albeit with non-trivial processing requirements; third, repeat sampling to track progression over time, or track response to interventions, is more feasible in a large population; and fourth, fluid biomarkers can provide insights into biological pathways, such as angiogenesis or inflammatory signaling.^151–153^ They also have potential limitations. Many vascular biomarkers are not specific to cerebrovascular cells,^153^ and may therefore be more of an index of overall cardiovascular status. This can introduce noise when trying to associate these markers with cognitive status or cerebrovascular brain injury.

Recent fluid-based protein candidates include: Platelet-derived growth factor receptor beta (PDGFRβ), placental growth factor (PlGF), vascular endothelial growth factor A (VEGF-A), interleukin-6 (IL-6), and matrix metalloproteinase-9 (MMP9), among others^151–153^ (Figure 2). Generally, these biomarkers point to two major biological pathways: inflammation and angiogenesis. More non-specific processes such as neuroaxonal injury (Neurofilament light; NFL) and astrocyte damage (Glial Fibrillary Acidic Protein; GFAP) have also been shown to be related to cerebrovascular injury-related cognitive impairment.^154,155^

**Figure 2.**
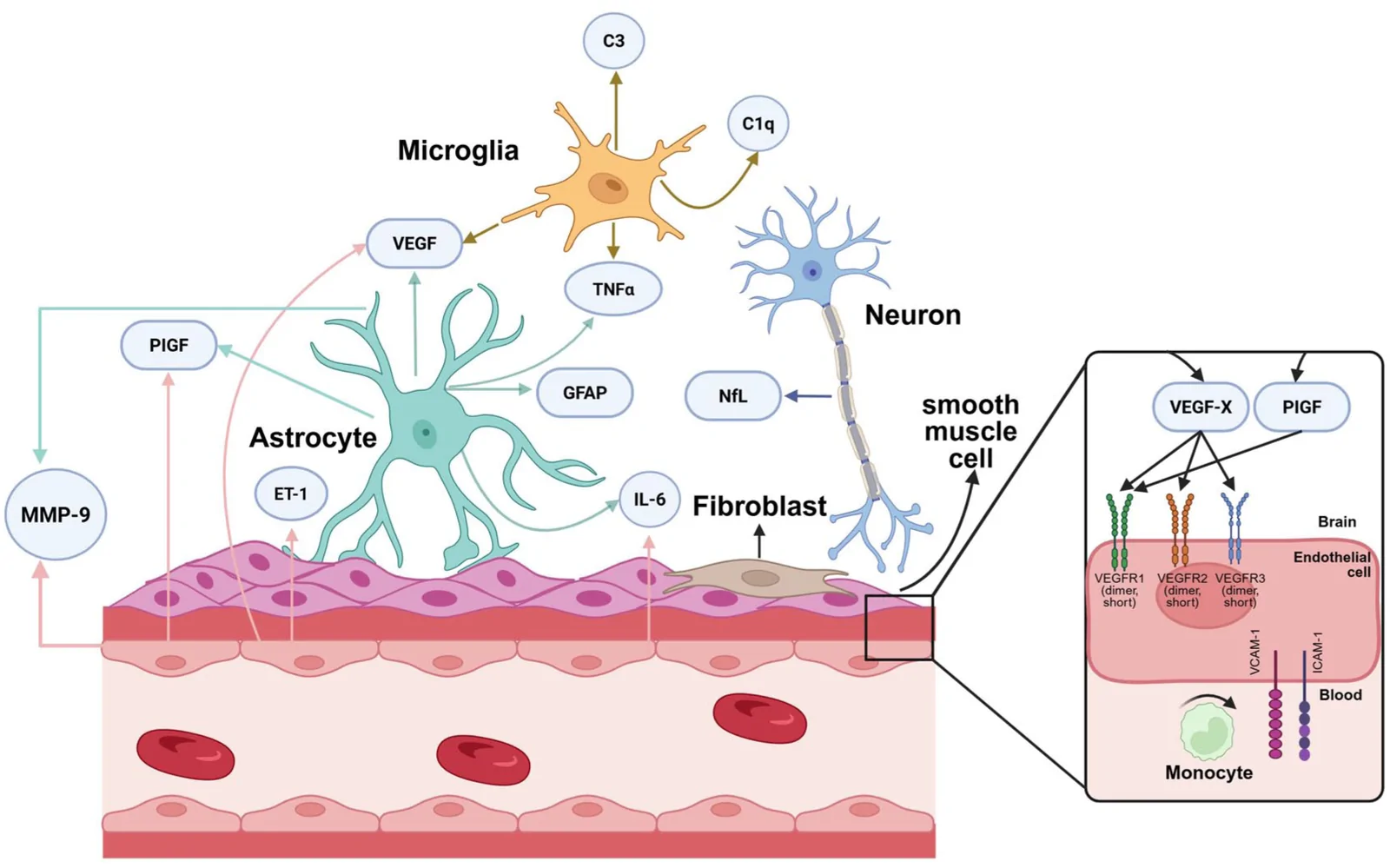
Schematic showing a cerebral small artery, with nearby parenchymal cells (neuron, astrocyte, and microglial cell). Examples of potential fluid biomarker molecules are shown.

A major obstacle to developing sensitive and specific biomarkers is the need for a gold standard. The candidate vascular biomarkers described have been related to radiological WMH load (WMH volume, or categorical Fazekas score) or to cognitive impairment likely due to cerebrovascular pathologies. Though generally regarded as an indicator of SVD, WMH can result from other vascular pathologies or even AD-related vascular pathology (likely mediated by CAA). Thus, associations with a particular fluid biomarker may depend on what brain pathologies are enriched in the test population. Lessons can be learned from the AD field, where fluid biomarkers advanced significantly only when an antemortem “gold standard” was available (in the case of AD, amyloid PET ligands).

One of the most robust fluid biomarkers for vascular disease identified to date is PlGF, an angiogenic regulator in the VEGF family.^156^ Although named after its primary role in placental vascularization, PlGF is expressed in the thyroid, fat, lymph nodes, heart, and brain. The PlGF molecule can dimerize with itself, or with VEGF-A, to modulate angiogenic processes, including endothelial cell survival, migration, vascular permeability, or to promote nonbranching angiogenesis. In the MarkVCID study, PlGF was assessed in three independent cohorts by three independent institutions and across those cohorts.^157^ This consortium-wide study demonstrated that in persons with a substantial WMH load, plasma PlGF increased in diagnostic accuracy from no cognitive impairment (CDR = 0; receiver operating curve, area under the curve, ROC AUC = 0.66), to MCI (CDR = 0.5; ROC AUC = 0.74), and performed best for those with dementia (CDR = 1; ROC AUC = 0.89).^157^ Several reports have since corroborated the association of PlGF with WMH and cognitive impairment in other cohorts.^158^

In addition to candidate proteins, novel tools such as vascular-specific extracellular vesicles, are providing alternative approaches to investigation of vascular disease in aging and across mixed neurovegetative disease states.^159,160^ This class of biomarkers, while promising, pose technical challenges that would need to be overcome for use in clinical trials and clinical practice. A growing body of literature is supporting the association of dysregulated angiogenesis with cognitive trajectories, with regulators of angiogenesis serving as promising biomarkers across neurodegenerative disease states.^157,161^

In the AD field, recent dementia-related blood-based biomarkers have shown substantial promise not only in research studies but also in clinical practice. These include Aβ40: Aβ42 ratio and total tau, and more recently phosphorylated tau species (p-tau181, p-tau217)^162,163^ and their brain-derived counterparts that are deemed likely to reflect brain AD pathology. Their relevance in cerebrovascular patients remains unclear, as these individuals have largely been excluded from population-based studies investigating AD-related biomarkers.^164^ Ongoing research efforts are beginning to address this gap by evaluating the role of these biomarkers in predicting cognitive and functional outcomes following stroke.^165,166^

## Interventions and clinical trials

Interventions targeting vascular disease in dementia may focus on primordial (vascular risk factor prevention), primary (treating vascular risk factors and subclinical vascular disease) or secondary prevention (treating VCI), see a listing of recent studies in Table 1. The best supported intervention for long-term reduction of dementia is control of blood pressure,^186^ reflecting the likely causative association between midlife hypertensive phenotypes,^187^ late-life chronic cerebrovascular disease,^188^ and subsequent all-cause cognitive decline.^19,186^ In SPRINT-MIND, intensive blood pressure control reduced WMH progression by a modest but significant amount (0.54 cm^3^ over 3.4 years)^189^ with a dose-effect across studies between achieved blood pressure control and rate of progression.^190^ This was associated with reduced cognitive impairment in individual studies^20,167,191^ and a 7–13% relative risk reduction, or 0.4% absolute risk reduction.^20,192^ This limited effect may reflect short-term treatment and a limited number of dementia cases in SPRINT-MIND. The ESO guideline^69^ also found in meta-analysis that WMH progression was more slowed with BP reduction to lower targets than with specific antihypertensive drugs, emphasizing that lower BP targets are better; however, as in SPRINT-MIND, the effect was very marginal for the extent to which BP had to be reduced. Optimization of treatment to the correct intensity, at the correct phase in life, may have a greater impact.

**Table 1.** Recent therapeutic trials in SVD and VCID.

Study	*N* (F/M)	Population; design	Main outcomes	Cognitive end point (Y/N)^a^
SPRINT-MIND^167,168^	9361 (3332/ 6029)	*Primary Prevention* Hypertensive adults ⩾ 50 years without diabetes or prior stroke; randomized, open-label with blinded endpoint adjudication; intensive systolic blood pressure (SBP) target < 120 mm Hg vs standard target < 140 mm Hg over mean 3.5 years of treatment; median cognitive follow-up of 5 years and extended to 7 years.	Primary cognitive outcome: adjudicated probable dementia (HR 0.83; 95% CI 0.67–1.04; P = 0.10). Secondary: mild cognitive impairment (HR 0.81; 95% CI 0.69–0.95; P = 0.007) and composite MCI or probable dementia (HR 0.85; 95% CI 0.74–0.97; P = 0.01). Maintained significant reductions in composite outcome and MCI over extended follow-up.	Y
ACE^169^	369 (191/ 178)	*Primary Prevention* Adults aged 65-85 year without dementia. Placebo-controlled randomized trial to 24 months of daily supplementation with 10 mg/day Equol or placebo. Follow-up of 24 months.	Primary: changes in arterial stiffness. Secondary outcome: Progression of WMH. Tertiary outcomes: cognitive decline on the Preclinical Alzheimer’s Cognitive Composite. Results: NR to follow November 2026 closeout.	Y
TREAT-SVDs^170^	75 sporadic SVD (20/55) 26 CADASIL (16/10)	Symptomatic sporadic SVD and CADASIL; multicentre open-label randomized crossover with blinded endpoint assessment; amlodipine vs losartan vs atenolol	Primary: CVR in normal-appearing white matter (BOLD-MRI hypercapnia) after 4 weeks of monotherapy. Result: no difference between amlodipine, losartan, and atenolol in sporadic SVD; in CADASIL, CVR improved with amlodipine and losartan vs atenolol.	N
OxHARP^171^	75 (16/59)	Symptomatic SVD after non-embolic cerebrovascular event with mild–moderate WMH; double-blind, randomized, placebo-controlled three-way crossover; 3 weeks each of sildenafil (up to 50 mg TID), cilostazol (up to 100 mg BID), and placebo; ⩾ 1 week washout.	Primary: MCA pulsatility index (TCD) sildenafil vs placebo (superiority) with non-inferiority vs cilostazol. Result: sildenafil did not reduce pulsatility vs placebo; key secondary: sildenafil vs placebo showed improved CVR (<0.048), reduced cerebrovascular resistance (P = 4.9×10^−8^), and increased perfusion (P < 0.008). Cilostazol vs placebo showed improved perfusion (P = 5.1×10^−5^)	N
ETLAS-2^172,173^	76 (20/56)	Patients with cerebral SVD and previous lacunar stroke or TIA (6 months to 5 years prior); randomized, placebo-controlled, double-blind, parallel phase II; tadalafil 20 mg daily vs placebo for 3 months.	Primary: feasibility (⩾90% drug compliance). Result: feasibility not met—⩾90% compliance 26/38 tadalafil vs 31/33 placebo (adverse events higher with tadalafil). Secondary: no clear differences in cognition (MoCA/IQCODE), BP, or MRI SVD markers overall; unadjusted per-protocol analysis suggested lower WMH volume with tadalafil (P = 0.054).	Y
PASTIS^174,175^	65 recruited 55 completed (15/40)	Older people with symptomatic SVD; phase II randomized, double-blind, placebo-controlled crossover; single-dose tadalafil (20 mg) vs placebo on two visits ⩾ 7 days apart; ASL-MRI.	Primary: subcortical CBF change by ASL after single-dose tadalafil vs placebo. Result: no significant increase in subcortical CBF overall; greatest (non-significant) effect within WMH; incidental BP reduction; no serious adverse events. No effect on cognitive end points.	Y
LACI-2^176^	363 (112/251)	Symptomatic lacunar stroke (SVD); PROBE randomized 2×2 factorial; isosorbide mononitrate (40-60 mg/day), ± cilostazol (200 mg/day) for 12 months.	Feasibility/safety trial. Result: ISMN and cilostazol were well tolerated and safe. Exploratory/secondary: combination ISMN + cilostazol associated with lower composite vascular/functional/cognitive outcome, lower dependence and cognitive impairment, and improved QoL (signals for phase III).	Y
DREAM (cilostazol for WMH)^177^	107 (51/56)	Community adults with confluent WMH; randomized double-blind placebo-controlled; cilostazol (100 mg BID) vs placebo for 2 years	Primary: WMH volume change over 2 years (cilostazol vs placebo). Result: WMH progression did not differ significantly between groups; secondary MRI/DTI, cognition, motor, mood measures broadly similar; acceptable safety profile.	Y
INFINITY^178^	199 (108/91)	Age ⩾ 75, systolic HTN with WMH; randomized parallel; intensive ambulatory SBP ⩽ 130 vs standard ⩽ 145 over 3 years.	Co-primary: WMH progression and gait speed. Result: intensive ambulatory SBP lowering reduced accrual of WMH vs standard (P = 0.03); no significant difference in mobility outcomes; major adverse cardiovascular events higher in the standard group; falls/syncope similar.	Y
ω-3 PUFA supplementation^179^	102 (62/40)	Older adults (⩾75) with WML burden + low ω-3; quadruple-blind RCT; EPA + DHA vs placebo for 3 years	Primary: annual WML progression. Result: ω-3 vs placebo did not significantly reduce WML accumulation. Secondary: DTI-FA decline not significantly different overall, but APOEe4 carriers had significantly less neuronal integrity breakdown (DTI-FA decline) with ω-3; adverse events similar.	N
PICASSO^180^	1512 (575/937)	Non-cardioembolic ischemic stroke with prior ICH or multiple microbleeds; randomized 2 × 2 factorial: cilostazol (100 mg BID) vs aspirin (100 mg); probucol (250 mg BID) vs no probucol	Primary: Major vascular events in ischemic stroke at high ICH risk. Result: cilostazol non-inferior to aspirin for composite cardiovascular events and did not reduce hemorrhagic stroke; probucol addition suggested benefit in reducing cardiovascular events.	N
AMCAD^181^	60 results pending (recruitment complete)	CADASIL; multicentre single-arm phase II; adrenomedullin infusion course (15 ng/kg/min for 8 h over 14 days)	Primary: change in frontal cortical CBF (ASL-MRI) and safety	Y
LOMCAD^182^	20 results pending	CADASIL with recurrent ischemic events; multicentre single-arm, historical-control comparison; lomerizine 5 mg BID for 24 months	Primary: symptomatic cerebral ischemic events over 24 months	N
T3-CAD-01 (HOV-12020 tocotrienols; NCT04658823) Unpublished	50 results pending (analysis ongoing)	CADASIL; randomized, placebo-controlled, double-blind (quadruple-masked) pilot/phase II; parallel 2-arm; HOV-12020 (palm tocotrienols complex) vs placebo for 24 months	Primary: incidence of clinical worsening events over 24 months (stroke, ⩾ 1 point mRS increase to ⩾ 2, or ⩾ 4-point decline in VADAS-Cog). Other outcomes include CDR-SB, MDRS, TMT, gait/balance, QoL.	Y
Oxiracetam + Physical Activity (post-stroke PSCI prevention)^183^	500 enrolled; 457 complete (109/348)	Post-stroke patients (⩾ 3 months) at high risk of post-stroke cognitive impairment (subjective cognitive decline); multicentre, randomized, double-blind, placebo-controlled; oxiracetam 800 mg BID vs placebo for 36 weeks; physical activity monitored by actigraphy.	Co-primary: change in MMSE and CDR-SB. Result: no significant between-group differences in MMSE change (oxiracetam + 0.13 ± 2.27 vs placebo + 0.27 ± 2.09; P = 0.49) or CDR-SB (−0.14 ± 0.70 vs −0.08 ± 0.80; P = 0.38); no interaction with physical activity. Exploratory: favorable trends in highly active oxiracetam participants.	Y
MINERVA (minocycline)^184^	44 (16/28)	Moderate-to-severe symptomatic SVD; phase II, randomized, double-blind, placebo-controlled; minocycline 100 mg BID vs placebo for 3 months; mechanistic PET/MRI (11 C-PK11195 PET + DCE-MRI).	Co-primary: microglial activation (11 C-PK11195 binding) and BBB permeability (DCE-MRI). Result: no effect on 11 C-PK11195 binding (RR 1.01, 95% CI 0.98–1.04) or BBB permeability (RR 0.97, 95% CI 0.91–1.03); serum inflammatory markers not affected.	Y
CONIVaD (nimodipine + choline alphoscerate)^185^	62 enrolled; 48 completed (25/23)	SVD with mild–moderate cognitive impairment; pilot, single-center, double-blind RCT; nimodipine 30 mg TID + choline alphoscerate 600 mg BID vs nimodipine alone; follow-up up to 2 years.	Primary cognitive outcome: no significant difference between groups; secondary outcomes also not significantly different. Safety acceptable, but adherence was low.	Y

NR: not reported; ASL: arterial spin labeling; BID and TID: twice daily and three times a day administration; CBF: cerebral blood flow; CI: confidence interval; CVR: cerebrovascular reactivity; DCE-MRI: dynamic contrast enhance magnetic resonance imaging; DTI: diffusion tensor imaging; FA: fractional anisotropy; HR: hazard ration; ICH: intracranial hemorrhage; IQCODE: Informant Questionnaire on Cognitive Decline in the Elderly; ISMN: Isosorbide Mononitrate; MCA: middle cerebral artery; MCI: mild cognitive impairment; MMSE: mini mental state examination; MoCA: Montreal Cognitive Assessment; RR: relative risk; TDC: transcranial Doppler; TIA: transient ischemic attack; WMH: white matter hyperintensities.

a Cognitive endpoint included as a pre-specified outcome? Tagged as Yes/No.

While the epidemiology is mixed regarding the potential cognitive benefit of particular antihypertensive classes and two large meta-analyses failed to find clear signals,^193,194^ large blood pressure-lowering trials and clinical practice frequently combine classes, making it difficult to tease out. The TREAT-SVDs trial failed to demonstrate improved cerebrovascular function with vasodilating antihypertensives (amlodipine, losartan, and atenolol) in sporadic SVD,^170^ although amlodipine did improve CVR compared with atenolol in CADASIL.^170^ Nonetheless, targeting cerebrovascular vasodilatory dysfunction remains a promising treatment target. The LACI-1 and LACI-2 trials^195^ have demonstrated improved cerebrovascular reactivity and improved cognition (as well as reduced recurrent stroke, less dependency, better mood and quality of life) at 1 year with isosorbide mononitrate, a nitric oxide donor, alone or in combination with the PDE3 inhibitor cilostazol, in SVD.^176^ Clinical benefit is now being tested in the phase III LACI-3 trial (ISRCTN44436843; target: 1300 patients in the United Kingdom). Cilostazol reduced recurrent stroke in Asian cohorts (CSPS, CSPS2, CSPS.com, CASISP, CATHARSIS, PICASSO), but was neutral with respect to cognitive outcomes^196^

In addition, a phase II trial in patients with MCI showed that cilostazol treatment was associated with an increase in plasma β-amyloid. This suggests enhanced clearance of Aβ from the brain, although no significant change in cognitive state (MMSE score) was detected over the 96-week treatment period.^197^ These findings indicate that alongside any potential cerebrovascular protective effects, cilostazol could be beneficial in VCI and also in mixed pathology in which vascular and AD mechanisms coexist.

The nitric oxide-cGMP pathway is the focus of Phosphodiesterase Type 5 (PDE5) inhibitor studies.^198^ This builds on a combination of observational data that PDE5i use is associated with reduced dementia,^199^ animal studies demonstrating improved vasodilatation, reduced tau accumulation and enhanced cognition, and causal genetic data from large datasets.^200^ Single-dose studies in SVD demonstrated only a trend for increased subcortical perfusion (PASTIS)^174^ and increased blood oxygen saturation in cortical small vessels (ETLAS-1).^201^ In the OxHARP trial, 3 weeks of treatment with sildenafil^171^ demonstrated improved cerebral perfusion, reduced cerebrovascular resistance and increased cerebrovascular reactivity, while 3 months of tadalafil in the ETLAS-2 trial improved cerebral perfusion.^172^ Similarly, PDE5 inhibition in early AD reduced both plasma p-tau181 and p-tau217 in a large phase II trial.^202^ The novel PDE5i mirodenafil is now being tested for cerebrovascular effects, alongside a multinational phase III efficacy trial in AD patients (POLARIS-AD; NCT05531526).

Additional promising pathways for primordial and primary dementia prevention may come from targeting arterial stiffness, which has been associated with incident dementia,^203,204^ often independent of blood pressure control. Structural aspects of arterial stiffness (elastin degradation, collagen cross-linking) may be impervious to BP control, even when the pulsatile force on the arterial wall is reduced.^205^ Aside from BP reduction, one avenue to treat arterial stiffness is selective estrogen receptor (ER) modulation. The ongoing Arterial stiffness, Cognition and Equol (ACE) trial^169,206^ targets ER through direct administration of a soy isoflavone metabolite, equol, which preferentially binds to ER-β and reduces total arterial stiffness over 12 months.^206^ The ACE trial will determine the effects of 24 months of equol on arterial stiffness, WMH load, and cognitive decline.

Targeting endothelial cell resilience may have benefits beyond cerebral perfusion. This is being tested in experimental crossover studies targeting the microglial protein TSPO in both cerebral SVD and AD.^207^ Targeting neuroinflammation may reduce brain injury in both SVD and AD. The recent phase II MINERVA trial of minocycline, targeting macrophage activation, did not demonstrate reduced neuroinflammation,^184^ possibly due to a sub-effective dose of the drug. A phase II trial of the Glucagon-Like Peptide-1 (GLP-1) agonist exenatide (GAPP; NCT05356104) is underway in Hong Kong (however, a recent large NovoNordisk trial of a GLP1 agonist in MCI-AD showed no benefit).

CADASIL is the most common hereditary SVD. Although there is currently an absence of established disease-modifying therapies, the condition may be amenable to vascular-targeted strategies.^13,208^ An ongoing trial (AMCAD) is evaluating the endogenous peptide adrenomedullin, known for its pro-angiogenic and vasodilatory properties, for its capacity to increase cerebral blood flow demonstrable using MRI arterial spin labeling.^181^ Likewise, a repurposing trial (LOMCAD) is testing whether lomerizine, a vasodilatory calcium channel blocker used for migraine prophylaxis, reduces ischemic stroke episodes in CADASIL.^182^ The TREAT-SVDs trial reported improved cerebrovascular function with amlodipine and losartan compared to atenolol in patients with CADASIL.^170^ These approaches, which directly target the cerebral vasculature, hold promise for pioneering therapeutic development in CADASIL.^209^ Findings from CADASIL are expected to provide insights that may ultimately be translatable to sporadic SVD more broadly.

Finally, treating vascular disease in dementia may profit from an environmental, non-pharmacological approach. Optimizing brain health in at-risk and mildly affected people through a lifestyle (“holistic”) approach aims to maximize and maintain cognitive function, and to reduce vascular drivers of disease. Trials including the cluster-randomized China CRHCP trial,^210^ MYB,^211^ POINTER,^22^ and FINGER trials,^212,213^ focused on individuals at increased risk of cognitive decline or dementia, demonstrating significant cognitive benefit from a multifactorial intervention.

Overall, despite the historic lack of interventions targeting vascular disease in dementia, the current expansion of clinical studies gives optimism (Table 1). It appears likely that interventions will move from the generic (blood pressure lowering) to more specific target pathways (ER modulation, neuroinflammation, nitric oxide signaling) and lifestyle interventions in the next decade, translating to a significant reduction in morbidity.

## Future challenges and opportunities

A review on this topic ten years hence will contain substantial new knowledge. This, combined with novel technologies, will enable this field to progress.^214^ Considering the current state of this field, and progress over the past decade, leads us to some key recommendations (Box 3). It remains a major hurdle to integrate routine recognition, assessment and treatment of vascular disease including effective vascular risk factor management in memory clinics and to implement routine cognitive assessment and management in stroke clinics. One aim of this review is to increase awareness of the inherent heterogeneity of VCID. Clinicians should note that a diagnosis of “VCID” is by itself not sufficiently informative on the nature of the underlying disease, prognosis, or treatment.^215^ Recommendation 1 (Box 3) offers guidance on how to refine the diagnosis.

**Box 3.** Recommendations.

Diagnosis and detection of VCID Recommendation #1. In clinical management: (1) obtain a comprehensive assessment of the cognitive deficit, as this directs care and support; (2) reach a specific etiological diagnosis of the nature and severity of the vascular disease, as this informs prognosis and directs secondary prevention; (3) consider if the cognitive deficit is sufficiently explained by the vascular disease, as this determines if other etiologies should be sought (as reflected in the VasCog-2-WSO criteria).^2^ Recommendation #2. To expand VCID markers, (1) include comprehensive vascular imaging biomarkers of VCID to accurately define the magnitude of VCID in AD, and to monitor the effects of shared vascular and dementia risk factor interventions; (2) expand neuroimaging-neuropathology correlative studies, to produce tractable imaging and neuropathologic markers of cerebral vascular disease in dementia. Establishing other types of VCID molecular markers (in plasma or CSF) is imperative. Recommendation #3. Refine, validate and adopt clinically meaningful diagnostic criteria that address mixed pathology in a balanced and pragmatic way across current sub-disciplines of AD and VCID. Understanding VCID biology to inform treatment Recommendation #4. In basic molecular studies, assess the tractability of molecular mechanisms of vessel pathology and tissue injury seen in neuropathological studies. Define how these relate to imaging features and how they contribute to cognitive impairment. Recommendation #5. For prevention of VCID, treat elevated blood pressure first according to current guidelines and reduce hypertensive vascular disease of the brain. Prioritize studies to investigate how amyloid-targeting therapies damage the blood vessels to cause ARIA-E and ARIA-H.^216^ Recommendation #6. To refine molecular targets in VCID, (1) characterize cell-type-specific gene expression and protein alterations in the brains of individuals with SVD, using further ‘omics studies on neuropathologically defined samples. (2) Specifically address white matter tissue, to decipher the cellular and molecular underpinnings of SVD (most single-cell and single-nucleus sequencing studies have focused on cortical regions). Accounting for the phenotypic heterogeneity and distinct spatial patterns of SVD subtypes will also be crucial.^217^ (3) Identify further repurposing drug candidates and novel therapies, building on molecular neuropathology, ‘omics and cell-type-resolved genetic risk architecture across SVD, AD, and cardiovascular traits.

Advancing treatment is clearly a key priority. As repurposing trials continue to capitalize on the merits of existing drug molecules,^176,218^ there are promises of new molecular entities (novel synthetic chemistry, molecular machines, artificial evolution).^214,219^ Multi-omic screening of DNA, RNA and proteins has identified multiple candidate genes, based on well-powered transcriptomic analyses, increasingly refined proteomics and careful interrogation of big genomic datasets. By contrast, platforms for lipidomics and polysaccharides remain under-developed and localized to a few expert labs (at least, in the dementia field).^220–222^ There may be qualitatively new dimensions of molecular medicine to be explored, based on lipid signaling or sugar coding.^223,224^

Deeper biological understanding of cellular behavior is progressing, with potential impacts on cerebrovascular disease in dementia. For example, a cell’s biological age (fascinatingly different from chronological age), cell senescence and autophagy all give hints that they may provide levers to control cell fate. A specific cellular context, highly relevant to dementia, is the neurovascular unit, where endothelial cells meet myocytes, fibroblasts and astrocyte end-feet. More informed approaches to treatment will likely come from a greater understanding of the cell biology of this brain vascular nexus.^225^ RNA atlases derived from human tissue biospecimens, alongside single-cell proteomics, are beginning to elucidate what happens in this unique domain in older humans.^123,129,133,136^ Ultimately, these advances will help with more precise differentiation of heterogeneous cerebrovascular contributions to dementia.

A number of disparate factors will enable more, and more informative and inclusive, clinical trials in VCID. First, a new source of clinical trial participants is likely to emerge from the increased uptake of amyloid-clearing therapies. Patients who are ineligible for anti-amyloid treatment, due to their vascular disease load, may welcome an alternative interventional drug.^226^ These individuals are likely to be eligible for dedicated VCID trials. Second, faster MRI acquisitions and low-field MRI scanners^227^ offer the potential to expand global capacity for the detection of relevant cerebrovascular pathology. While low-field instruments provide only low-resolution images, they have the advantages of low cost, portability and no requirement for increasingly rare liquid helium. They are being considered for clinical use, particularly in low- and middle-income countries, as well as locations in high-income countries where access to fixed MRI systems is challenging (such as community-based memory services). As brain MRI has become central to any clinical investigation of brain vascular disease, low-field machines offer a genuine opportunity to expand access to diverse populations to a VCID-related trial. And third, it seems inevitable that artificial intelligence will change the logistics of VCID research at many levels. In terms of brain imaging in clinical populations, possible benefits of the AI revolution include faster acquisition and analysis, greater homogeneity across centers, and possibly novel patterns (more easily detected by a machine than by a human).
